# Task-Technology Fit of Artificial Intelligence-based clinical decision support systems: a review of qualitative studies

**DOI:** 10.1186/s12911-025-03237-8

**Published:** 2025-10-28

**Authors:** Cathleen S. Parsons, Anneke Zuiderwijk, Nic A. Orchard, Jacobien H. F. Oosterhoff, Mark de Reuver

**Affiliations:** 1 Department of Engineering Systems & Services, Faculty of Technology, Policy and Management, Delft University of Technology, Jaffalaan 5, Delft, 2628 BX Netherlands; 2 Department of Orthopaedic Surgery, University Medical Centre Groningen, University of Groningen, Groningen, Netherlands

**Keywords:** Artificial intelligence, Clinical Decision Support System, Task-Technology Fit, Human-AI collaboration, AI adoption, Attitude of health personnel

## Abstract

Machine learning algorithms show promise in assisting clinical decision-making; however, only a few have been successfully implemented in practice. To bridge this gap, it is essential to analyse the clinicians’ perspective on the compatibility of Artificial Intelligence-based clinical decision support systems (AI-CDSSs) with their clinical tasks. We therefore conducted a literature review of 21 empirical qualitative studies that examined the interaction between health professionals and AI-CDSSs. We synthesised the research through the lens of the Task-Technology Fit (TTF) model, analysing task, technology and individual characteristics of AI-CDSS applications, to identify design elements that are (mis)aligned with clinicians’ needs. Three key findings emerged from our analysis. First, clinicians often expressed scepticism about the clinical judgements of AI-CDSSs, particularly questioning the system’s ability to compete with clinical expertise in the absence of contextual information. Users valued AI primarily for specific strengths, such as identifying trends in patient trajectories, consolidating large datasets and pattern recognition, and comparing similar patient cases, but were hesitant to rely on it for clinical decisions. Second, actionability emerged as a desired characteristic of AI-CDSSs. For instance, clinicians particularly appreciated features of AI-CDSSs that enabled them to explore how different clinical actions might influence outcomes, as well as Explainable AI for identifying modifiable variables that impacted prediction scores, allowing them to take informed action. Third, we identified various ways AI-CDSSs could be used in clinical practice, including for patient prioritisation, patient monitoring, care acceleration, risk communication and workflow efficiency. In essence, AI-CDSSs functioned either as an alert system, preventing oversights, or as a tool for more informed decision making. Our analysis challenges the assumption that AI-CDSSs add little value when clinicians disregard its predictions, as it frequently prompts them to critically reassess their judgments through additional testing, consultation with colleagues, and other actions. Overall, our findings underscore the importance of an in-depth understanding of how AI-CDSSs are used in clinical practice. To optimise for effectiveness, the design of AI-CDSSs should prioritise supporting clinicians’ cognitive processes and information needs. This approach ensures that we move beyond the hype, focusing on the responsible integration of AI-CDSSs, and ultimately enhancing patient care.

**Supplementary Information:**

The online version contains supplementary material available at 10.1186/s12911-025-03237-8.

## Introduction

Artificial intelligence (AI) in healthcare has the potential to transform the way healthcare professionals make diagnoses, therapeutic and prognostic recommendations [1, 2]. By leveraging multi-source data and machine learning, AI-based clinical decision support systems (AI-CDSSs) personalise patient care and can improve healthcare services, thereby reducing healthcare costs [3]. As healthcare organisations modernise their digital infrastructure, the increasing availability of healthcare data is expected to accelerate the development of AI-CDSSs, fundamentally reshaping healthcare delivery [4].

Despite the significant promise of AI-CDSSs in healthcare, empirical evidence of its clinical effectiveness remains limited. For example, two systematic reviews of randomised controlled trials (RCTs) have concluded that the impact of AI-CDSSs on clinical outcomes has often been modest [5, 6]. These findings suggest that the development of an accurate AI tool alone is insufficient to achieve value in real-world settings. Implementation science has already emphasised the need for a comprehensive systems perspective that considers multiple dimensions - such as acceptability, feasibility, and fidelity - which collectively determine an intervention’s impact [7].

Recent research [8] revealed that RCTs on AI-CDSSs still tend to adopt a narrow focus, leaving various dimensions described by Proctor et al. [7] unaddressed. One such underexplored implementation outcome is ‘appropriateness’, which refers to the compatibility of AI-CDSSs with clinical tasks and whether clinicians found the tools useful [8]. Since AI-CDSSs are, by definition, designed as support tools, their performance and clinical value are intrinsically tied to clinician interactions and perceived functionality.

This paper aims to synthesise qualitative research that describes the clinician’s perspective when interacting with AI-CDSSs. To this end, the Task-Technology-Fit (TTF) model is applied [9]. Previous literature reviews have explored clinicians’ perspectives on AI-CDSSs from various angles. For instance, Lambert et al. [10] investigated clinician acceptance using the Unified Theory of Acceptance and Use of Technology (UTAUT). Other reviews from clinician perspective have taken broad approaches covering effectiveness, outcomes, and costs (e.g., [11]) or concentrated primarily on technological aspects without considering task-related factors (e.g., [12, 13]). Wang et al. [14] provided a comprehensive clinician-centered review synthesising user needs and adoption challenges and design implications, though without specifically addressing the interplay between task demands, system capabilities, and user characteristics.

By adopting a TTF perspective, this literature review addresses a niche by offering a unique design-centric approach to enhance the integration and adoption of AI-CDSSs in clinical practice. For our study, we distinguish AI-based CDSSs from conventional CDSSs by defining AI as machine learning approaches that enhance their task performance through exposure to additional data [15]. We align with Berente et al. [16]’s perspective that AI represents a sociotechnical “moving frontier” with evolving boundaries. Following prior work in clinical prediction modelling [17], we exclude conventional statistical methods such as logistic regression, from machine learning approaches. This scope allows us to focus on the transparency and usability challenges associated specifically with AI-based CDSSs. Moreover, we restricted the review to AI-CDSSs that analyse tabular data, excluding imaging- and Natural Language Processing-based CDSS whose fundamentally different technological mechanisms and data representations would compromise synthesis in our TTF analysis.

As the first step in this process, the background section of this paper will provide an overview of the TTF model, outlining its core concepts and relevance to this study. Results retrieved from the included articles will be then categorised under the model’s core concepts, with implications explored in the discussion section.

## Theoretical model Task-Technology-Fit

A Task-Technology Fit, as defined by its conceptual architects Goodhue and Thompson [9], is *‘the degree to which a technology assists an individual in performing his or her portfolio of tasks’* (p. 216). According to the TTF model, this fit depends on the alignment of (1) task specificities, (2) characteristics of the individual, and (3) technological features. TTF therefore stresses that we should not assess the performance of a technology without considering its context of use [9]. In their paper, Goodhue and Thompson [9] situated the TTF within the broader framework of the Technology-to-Performance chain model, which includes additional precursors to utilisation. By placing performance impact as the end outcome and not utilisation, the authors emphasise responsible use of technology. A singular focus on increasing technology utilisation could undermine performance [9]. This issue is particularly relevant in the context of AI-CDSSs, where overreliance - accepting false negative or false positive predictions - becomes problematic. While literature indicates that AI-CDSSs are being underused [5, 18], overreliance on AI-CDSS advice may lead to the acceptance of inaccurate results [19]. False positive and false negative recommendations can misguide clinicians, resulting in significantly lower performance [19]. Therefore, TTF has a preference over other task technology fit models, such as the Ammenwerth, Iller and Mahler [20] ‘Fit between Individuals, Task, and Technology ‘ (FITT) model, which has not been theoretically placed within a broader context as the TTF is in the Technology-to-Performance chain model.

Furthermore, in their meta-analysis of research that has applied TTF, Cane and McCarthy [21] concluded that, compared to the Technology Adoption models, TTF is particularly well-suited for generating design-oriented recommendations, as it explores the preconditions for perceived usefulness. For instance, one study [22] has used the TTF model for designing a mobile clinical support decision tool. A TTF analysis can thus provide valuable insights for the implementation outcome ‘appropriateness’^1^, which is currently less explored compared to ‘acceptability’^2^ [8]. The TTF has been applied in many IT domains, including education, health care and business fields [23, 24]. TTF of health technologies specifically has been assessed quantitatively (e.g. [18, 25–27]), qualitatively [28] or with mixed method (e.g. [22, 29, 30]). Studies on subject related topics, such as task-technology fit of an mHealth app [28] or clinical decision support systems [18, 22], have already demonstrated the usefulness of this model for gathering relevant insights. Of particular relevance is a survey of 247 clinicians conducted by Zheng et al. [18]. Their findings confirmed that the task-technology fit of CDSSs, through its effects on performance expectations and reduced perceived risk, positively influence clinicians’ intention to use CDSSs. Although personal characteristics were not considered, technological characteristics had a substantially stronger influence on TTF than task characteristics [18].

In this review, all core concepts of TTF model are examined. Goodhue, who conceptualised the TTF, stated that user evaluations are the preferred method for determining task-technology fit [31]. Accordingly, we framed the following research questions from the perspective of clinicians interacting with AI-CDSSs:

1. **Technology characteristics (**section “Technology characteristics: limitations and needs”**)**: *Which specific features or functionalities of AI-CDSSs do clinicians perceive as increasing or decreasing their fit with clinical tasks?*
2. **Task characteristics (**section “Task characteristics: opportunities and challenges”**)**: *Which clinical task characteristics create opportunities or challenges for the perceived TTF of AI-CDSSs?*
3. **Individual clinician characteristics (**section “Individual characteristics: competencies and cognitive frameworks”**)**: *How do differences among clinicians affect their perception of the TTF of AI-CDSSs?*
4. **Perceived Task-Technology-Fit (**section “Perceived Task-Technology-Fit”**)**: *In what way do clinicians integrate AI-CDSS into their clinical decision making?*

Using the TTF model, the synthesis of qualitative data in this study focuses on the perceived usefulness of AI-CDSS features and functionalities in relation to clinical tasks and highlights any misalignments that may explain the low uptake in clinical practice.

## Methods

### Scope

The goal of the literature search was to identify all studies that have qualitatively and empirically assessed the interaction between the clinical user and an AI-CDSS. Because of our assumption that the reasoning processes and needs of clinicians significantly differ per type of AI, we focused on AI-CDSSs using tabular data instead of those based on imaging or text data via computer vision or natural language processing. Originally, we intended to include only studies that evaluated AI-CDSSs that had been implemented in clinical practice; however, due to a scarcity of such studies, we included all types of qualitative empirical research with AI-CDSSs.

### Databases search

Our search strategy, in collaboration with a librarian, included a combination of multidisciplinary and specialized databases: Web of Science (core collection) (multidisciplinary), Dimensions (multidisciplinary), PubMed (life sciences and biomedical), IEEE Xplore (computer science). ArXiv preprints were included in Dimensions search queries to capture recent developments. Databases were searched up to 9th of July 2024, with no date restrictions applied.

Three domains of keywords were combined using ‘AND’; within these domains, terms were linked with ‘OR’. The first domain encompassed terms related to machine learning (ML), the second to decision support tools, and the third to both qualitative and empirical studies. To exclude studies on AI- CDSS for imaging data, ‘NOT’ was added to filter out references related to radiology. For searches in non-medical databases, a fifth domain specifying the health domain was included. Detailed description of search terms can be found in Supplementary Material 1.

### Eligibility criteria

The inclusion criteria were defined as follows: an empirical study design, the use of a clinical decision support tool based on AI, and qualitative evaluation of either implemented or simulated AI-CDSS. Exclusion criteria were specified as: non-ML prediction model (e.g., logistic regression), AI-CDSS primarily consisting of computer vision or natural language processing algorithms, mental healthcare AI-CDSS, quantitative research design, lack of full text, conference abstracts, book chapters and languages other than English or Dutch.

Titles and abstracts were independently screened by two reviewers (CSP and NAO) using Rayyan software [32]. To refine and calibrate their screening methods, a pilot screening of 10 articles was conducted. Full-text articles were then assessed for eligibility by the first author (CSP).

### Data extraction

The following data were extracted from the included studies: first author, year of publication, country, title, AI-CDSS’s outcome, participants included, implementation phase and qualitative results (including supplementary material for quotes). The implementation phases were categorised as: ‘Mock AI’ (not yet based on real data), ‘Test AI’ (based on data but with vignette-based evaluation) and ‘Implemented AI’ (deployed in clinical practice). Qualitative results were analysed using the qualitative analysis software Atlas.TI (version 24). A descriptive overview of the extracted information from each included study is available in Supplementary Material 2.

### Data analysis

First, we familiarised ourselves with the extracted data and applied a priori coding using the core components of the TTF model as organising categories: technology characteristics, task characteristics, individual characteristics, and perceived task-technology fit. Within these categories, the first author (CSP) conducted inductive coding of the qualitative findings to derive subthemes (e.g. ‘personal_AI literacy’ or ‘technology_customisation’). We followed the six-phase approach described by Braun & Clarke [33, 34], which offers a flexible yet systematic framework for identifying and integrating patterns across heterogeneous qualitative studies. We used a single-coder analysis approach to ensure analytic depth and coherence in the iterative coding process. This approach aligns with the reflexive thematic analysis (RTA) premise that “meaning and knowledge are understood as situated and contextual, and researcher subjectivity is conceptualised as a resource for knowledge production” [35, pp. 334–335]. We therefore make explicit that the first author’s multidisciplinary background in medicine and in data science in population health management inevitably shaped coding and interpretation. The analysis involved developing three substantially revised versions of the analytical narrative. Each version refined our interpretation of how task, technology, and individual characteristics interact to shape the use of AI-CDSSs, progressing from broad, general themes to a more coherent, coding-level narrative. The final coding scheme is presented in Supplementary Material 1.

The following section presents the results of this thematic analysis. Due to the heterogeneity of case studies and for readability, clinicians’ perspectives are synthesised without isolating them by level of evidence (e.g., evaluation of implemented, experimental, or hypothetical AI-CDSS features) or differentiating between direct quotations and authors’ interpretations.

## Results

### Overview of included articles

**Fig. 1:**
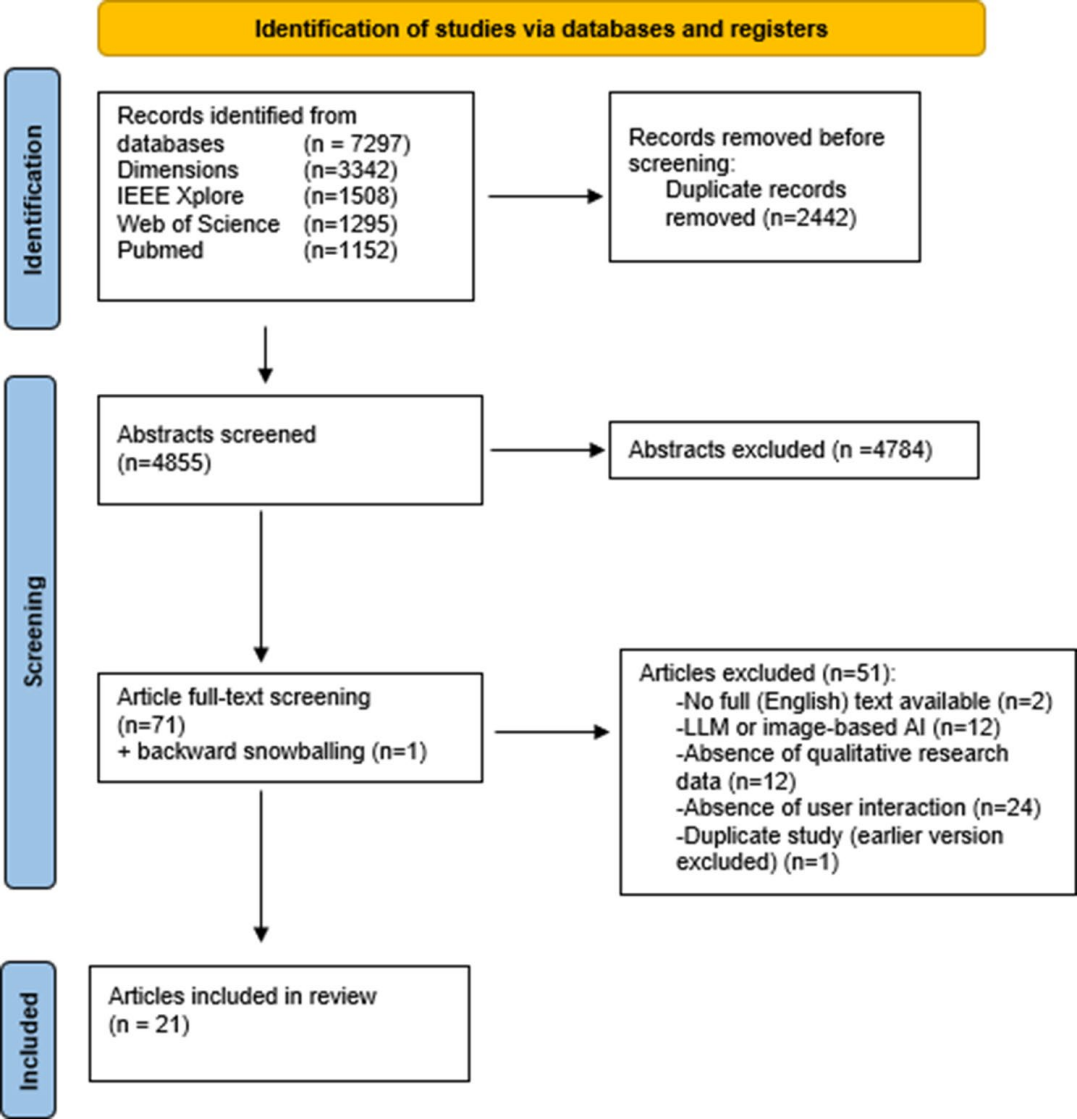
Flowchart of study inclusion and exclusion. Abbreviations: n = number, LLM = large language model, AI = artificial intelligence

A total of 7297 studies were identified and 4855 unique studies remained after duplicate removal. After the title/abstract screening, 72 studies were deemed potentially relevant. Of these, 21 met the inclusion criteria (Fig. 1).

Table 1 provides an overview of the 21 case studies where clinicians interacted with AI-CDSSs. Nearly all included studies were published after the year 2020 (*n* = 20), and most were studies conducted in the USA (*n* = 11). The maturity phase of AI-CDSSs was predominately in test AI (*n* = 12), followed by implemented AI (*n* = 8), and lastly Mock AI (*n* = 2).

**Table 1:** Characteristics of included studies (*n* = 21)

# ID	Ref	First author (Year)	Country	AI-CDSS outcome	Maturity of AI-CDSS	Study participants
#1	[36]	Abdulaal (2021)	UK	Probability of death during the current hospital admission for COVID-19 patient	Test AI	31 physicians
#2	[37]	Abraham (2023)	USA	Perioperative risk predictions for acute kidney injury, delirium, pneumonia, deep vein thrombosis, and pulmonary embolism	Test AI	17 clinicians
#3	[38]	Anjara (2023)	Spain	Stratification lung oncology patients based on post-treatment complication risks, predicting chances of patient’s relapse	Test AI	10 oncologists
#4	[39]	Choudhury (2022)	USA	AI–based blood utilisation calculator (BUC) for red blood cell transfusion	Implemented (> 1 year)	10 clinicians
#5	[40]	Fritz (2024)	USA	Probability postoperative death, acute kidney injury, respiratory failure, and myocardial infarction.	Mock AI	25 anaesthesiologists
#6	[41]	Henry (2022)	USA	Risk alert sepsis	Implemented (> 6 months)	20 clinicians
#7	[42]	Jauk (2021)	Austria	Delirium risk stratification tool	Implemented (> 7 months)	15 clinicians
#8	[43]	Jin (2020)	China	Diagnosis risk and treatment outcome analysis (estimation of the most influential treatment)	Test AI	7 physicians (1 based in USA)
#9	[44]	Liu (2023)	USA	Vancomycin dosing	Test AI	13 critical care pharmacists
#10	[45]	Matthiesen (2021)	Denmark	Predicting ventricular tachycardia and ventricular fibrillation (VT/VF) within 30 days	Test AI	7 electrophysiologists
#11	[46]	Naiseh (2023)	UK	Classifying chemotherapy prescriptions as confirmed or rejected + five types of XAI	Mock AI	16 clinicians (physicians and pharmacists)
#12	[47]	Panigutti (2022)	Online	Binary prediction of acute myocardial infarction	Test AI	28 health care professionals (including 25 clinicians)
#13	[48]	Payne (2024)	USA	Early Warning Score Sepsis	Test AI	12 clinicians
#14	[49]	Samhammer (2022)	Germany	Risk prediction of infection and graft loss in the next 90 days.	Test AI	14 nephrologists
#15	[50]	Sandhu (2020)	USA	Sepsis risk within the subsequent 4 hours	Implemented (> 4 months)	15 Emergency Department clinicians
#16	[51]	Schwartz (2022)	USA	Risk of in-hospital deterioration	Implemented (> 3 months)	17 clinicians
#17	[52]	Sivaraman (2023)	USA	Recommendation of an action state of 5 possible levels of Intravenous fluids and 5 vasopressor dosages	Test AI	24 Intensive Care Unit clinicians
#18	[53]	Wang (2021)	China	Recommendation of most likely diagnoses	Implemented (> 6 months)	22 clinicians (incl. 4 traditional Chinese medicine professionals)
#19	[54]	Yang (2019)	USA	Supporting the decision to implant ventricular assist device (VAD) by visualizing the patient outcome predictions, including life expectancy, estimated time until right heart failure, and likely cause of death	Test AI*	17 clinicians
#20	[55]	Yoon (2024)	Singapore	Diabetes medication prescribing recommendation and diabetes complication risk prediction	Implemented (> 4 weeks)	13 clinicians
#21	[56]	Zhang (2024)	USA	**Previous AI-CDSS**: alert for sepsis **New AI-CDSS**: patient’s current and in the next 4 hours sepsis risk score, including prediction uncertainty, and actionable suggestions to reduce such uncertainty	**Previous AI-CDSS**: Implemented **New AI-CDSS**: Test AI	6 clinicians

Overview of included studies. Abbreviations: AI-CDSS = Artificial Intelligence-based Clinical Decision Support Systems, USA = United states of America, UK = United Kingdom. *Operational but not yet data-driven (simulated)

### Analysis of Task-Technology-Fit of AI-CDSSs

The section first presents the technology-related characteristics that enhance or constrain TTF (4.2.1), followed by an exploration of how task characteristics (4.2.2) and individual characteristics (4.2.3) affect TTF. We then examine how clinicians integrate AI-CDSS into their decision-making processes (4.2.4) and conclude with a visual summary of the overall results (4.2.5).

#### Technology characteristics: limitations and needs

Despite the variety of AI-CDSS predictions, spanning from delirium to sepsis, clinicians have encountered similar limitations when applying these tools in clinical practice. In multiple case studies, clinicians questioned the usefulness of percentages as outputs, a standard practice in data science (#7, #10, #19, #20, #21). Beyond their role in prioritising urgency *between* patients, precise percentages often posed interpretive challenges for clinicians when assessing individual patient risk scores:

So I’m not really sure what its goal is, but I can tell you that most of us ignore it (the sepsis risk score) because it has not proved helpful to what we do next. (Emergency Room physician, #21)

Therefore, in one case study, risk predictions expressed as percentages were replaced shortly after implementation with a three-tier visual risk categorisation (#7). This approach was considered more actionable, helping clinicians to identify patients near the thresholds between risk groups and in need of extra care.

Multiple clinicians highlighted the need for AI recommendations to be directly applicable to their tasks (#2, #3, #5, #10, #16, #20, #21). Case studies (#4, #9, #17, #20, #21) involving AI-CDSS that provided specific task recommendations - such as drug doses, IV fluid, lab tests, red blood cell transfusion - elicited generally positive responses from clinicians, particularly in comments about perceived usefulness and the degree to which the systems were integrated into decision-making, compared to other case studies. However, overly granular percentage differences (#20) or impractical recommendations, such as excessively small doses (#17), diminished clinical relevance:

I’ve never ordered such a small dose of fluids… To me, that’s like sprinkling water on her. (Attending physician Intensive Care Unit, #17)

Furthermore, AI-CDSSs that were overly directive in their recommendations created unease among clinicians. This was highlighted in a case study where one clinician remarked, “seems like the system was designed to replace doctors” which limited the system’s use in clinical practice (#18).

To enhance task relevance, clinicians often expressed a desire to understand the most influential variables driving prediction scores (#2, #3, #6, #8, #9, #10, #13, #14, #15, #16, #17, #18, #21). They consistently highlighted that explainable AI (XAI) is primarily valuable when it offers insights into variables they can directly influence in their clinical practice. In contrast, they found less value in XAI outputs that focused on, for instance, reactive physiological variables that have minimal impact on treatment (#2), uncontrollable factors such as age (#11), incomparable patient cases (#3), or medically unrealistic counterfactual explanations (#11). Understanding the relationships between these modifiable variables and risk predictions enables clinicians to take actionable steps in patient care:

[The current prototype is] still kind of missing… the action item, right? What should the blood pressure be to decrease risk, right? Or if this patient is already mechanically ventilated, what should the CO2 be to decrease risk? (Anaesthetist, #2)

Moreover, some clinicians stressed the need for underlying variables that drive predictions to have causal relationships rather than mere statistical associations to derive clinically meaningful interpretations (#11, #14, #19). Although clinicians expressed high standards for explainability, its complete absence was viewed unfavourably in the one case study that lacked any form of XAI. In this example (#9), clinicians demonstrated low adherence to vancomycin dosing recommendations, despite the actionable nature and well-received purpose of the AI-CDSS. This was attributed to the “black box” nature of the recommendations (#9).

A second limitation frequently mentioned in interviews, was AI-CDSSs lack of access to key information clinicians rely on, such as free-text notes (#14), bedside observations and patient interactions (#6, #14, #17, #18), and contextual factors such as insurance policies (#18). One participant in #17’s study highlighted this gap:

At the bedside, I would acquire one or two pieces of reliable, better-quality data than the algorithm has available. And then I would use that to make my decision […] It’s not fair to ask an algorithm to make a prediction as reliable as that, because it doesn’t have access to that. (Attending physician Intensive Care Unit, #17)

Additional concerns included poor data documentation quality (#8, #13), adjustment of medical device settings (#10), evolving medical knowledge and practices (#1, #8, #11), and delays or unavailability of critical data (#16, #21). This affects task usability, especially in instances where healthcare professionals fail to document information correctly:

There are many times vital signs are documented incorrectly, such as a temperature of 20… AI is going to tell me there’s a problem. That’s where the human component comes in… It appears AI spits out numbers saying ‘Go deal with it.’ That’s when I don’t really trust the system. I still have to go and look at it, which defeats the purpose of the system. (Rapid Response Team clinician, #13)

Despite these limitations, AI-CDSSs were acknowledged for its unique capabilities in supporting clinicians in their clinical practice. Clinicians valued how AI-CDSSs helped to process the large volume of parameters and data (#6, #14, #16, #19) and facilitated access to data sources that clinicians often lack time to review, such as nursing notes (#16):

[N]ot that it takes over my work, but that it helps me to record everything […] because I am no longer able to record all the data that is collected. It is difficult for me to look through a laboratory with 35 parameters and to look at every value and somehow not miss anything. And that helps me to somehow make a correct assessment or to point things out to myself (Junior physician, #14)

A second strength often mentioned, was AI-CDSS’s ability to detect subtle changes in patterns and trends that clinicians have difficulty noticing (#5, #13, #16, #20), which could help to provide timely care:

Maybe the algorithm’s better at like kind of like nudging us to just like readdress some things that maybe are changing minutely day to day, so we may miss if we’re if we’re not, like, really aware of the trend. (Physician, #16)

Clinicians particularly valued visualisations that displayed timelines of patient trajectories, improving prediction comprehension (#13, #20, #21). However, early predictions occasionally created friction, as clinicians tended to reject AI-CDSSs in the absence of clinical validation, particularly when concerned about potential iatrogenic harm (#6, #15, #16). Due to this friction, one case study adjusted the timing of alerts to align with real-time clinical assessments (#6). #17 noted how a 4-hour predictive window induced clinical hesitation and delayed decision-making in high-stakes cases, and thus potentially diminishing the system’s prognostic utility. Similar observations were made by a nurse whose role was to alert Emergency Department physicians to patient risk based on the Sepsis Watch tool (#15):

I think a big part of people not understanding [Sepsis Watch], including the ED [emergency department] doc, is if vitals are stable. We’re not gonna treat because they look stable. I know but we’re trying to catch it before it’s unstable. And that’s the biggest piece people don’t get…fact that it’s predictive like, hammering that in will help people see…we’re trying to prevent the decline. (Rapid Response Team nurse, #15)

Thirdly, AI-CDSSs provide clinicians with valuable tools to explore the impact of different clinical actions. Access to similar historical cases to compare treatment and diagnosis strategies was generally well-received (#8, #11, #14, #17, #18):

Seeing the different outcomes to those decisions in a similar case, I think is…the most convincing to change your clinical decision making (Attending physician Intensive Care Unit, #17)

However, clinicians also raised concerns that such comparisons might lead users to mimic less experienced clinicians and their errors in clinical management (#17). Additionally, another interviewee noted that the concept of similarity between patients is not straightforward, and clinicians need to consider how it is exactly defined before application (#11).

Beyond analysing historical cases, adjustable predictions were seen as another valuable approach to support clinical decision-making (#3, #8, #17, #19, #21). #8’s case study demonstrated that the ‘treatment outcome analysis’ feature, where the impact of different treatments was compared, was highly valued by clinician users, while #21’s case study showed strong clinician interest in understanding the predictive impact of different laboratory tests. Clinicians expressed additional preferences for customisation, including control over variables used in score predictions (#14), the ability to define similarity metrics (#11), and flexibility in specifying features for counterfactual analyses (#11). Furthermore, clinicians emphasised the importance of being able to adjust the prediction score thresholds to align with local workflows, prioritisation rules, individual clinician treatment strategies and patient populations (#4, #10, #17, #21).

If you set them too low, you’ll get way more alerts than might be clinically present. And you’ll likely get fatigued and potentially [ignore alerts]. If you set them too high, you make it unlikely that any of these would ever be prevented, because you’re apt to just… have them develop. (Anaesthetist, #2)

In conclusion, clinicians identified actionable risk scores, actionable XAI, scenario cross-analysis, trend detection, and customisation as desirable AI-CDSS features for clinical practice. However, limitations such as restricted access to key information, concerns about data quality and reliability, and the timing of recommendations reduced the applicability of AI-CDSSs in clinical practice.

#### Task characteristics: opportunities and challenges

Clinicians identified several application areas where AI-CDSSs could provide valuable assistance to clinical practice: care acceleration and patient prioritisation, operational efficiency, risk communication and by adding objectivity to decision-making processes.

Most often, clinicians expressed interest in or had experience using AI-CDSSs to identify opportunities for early intervention and care acceleration to improve patient outcomes (#1, #2, #3, #6, #7, #10, #13, #14, #15, #16, #21). Examples included were more rapid transfer to ICU (#1, #13, #15), earlier treatment for delirium patients (#7) and more intensive interventions for ventricular tachycardia and ventricular fibrillation (#10). Furthermore, AI-CDSSs were considered valuable for patient prioritisation, where risk scores could guide clinicians’ attention allocation, particularly in time and resource constrained settings (#2, #6, #10, #13).

The EWS triggers my mind to investigate a chart and see what’s going on and identifies patients to focus on, evaluate first, and decide if I need to intervene. (Rapid Response Team clinician, #13)

Regarding operational efficiency and workload reduction, an AI-CDSS demonstrates multiple benefits: it accelerates clinical processes through rapid decision-making (#6, #7, #8, #10, #13, #14, #16, #21), performs calculations and personalises treatments doses (#4, #17) and helps alleviate cognitive burden in an environment where clinicians feel “bombarded with clinical information” (#6).

In a fourth application area, AI-CDSSs facilitate risk communication between different healthcare professionals, thereby helping to co-ordinate care (#1, #2, #19). When used in discussions with patients and their families (#1, #2, #20), effective risk communication improves treatment adherence (#20) and helps intervene on contributing factors to risk estimations preoperatively (#2). Additionally, two case studies noted how AI-CDSSs strengthen the position of nursing staff by providing evidence-based support for their clinical judgments, such as their recommendations for extended hospitalisation or medical procedures (#12, #19):

The doctors in our acute medical department are very keen to discharge patients home; leaving nurses in a difficult predicament when we don’t agree with their decision making. A tool such as this, could help nurses to justify their reasons for keeping a patient in hospital or to use cardiac monitoring vs. not monitoring. (Nurse, #12)

As an overall tool, AI-CDSSs have the potential to enhance clinical decision-making by introducing objective analysis. In #17’s study, the participants who consistently incorporated AI recommendations were those who emphasised the objectivity of data. This objectivity proves particularly valuable in several scenarios: when handling controversial cases with conflicting opinions (#14), during emotionally charged decisions and conversations (#13, #14, #19), and in complementing clinicians’ intuitive assessments such as “gestalt” and “gut feelings” (#5, #13). Thus, AI-CDSSs help to mitigate any human error (#13), reduce subjectivity (#13, #14, #19), maintain attention despite fatigue (#8, #16) and distractions (#20), while limiting effects of potential personal bias towards patients and recency bias in clinical judgment (#10, #19).

I feel like I have that kind of gestalt if someone’s going to be okay, but it’s nice to see the numbers. It’s really nice to see the numbers. (Attending anaesthetist, #5)

When I really like this patient, really want to help him or her, it sometimes helps to get a more factual view. (Unspecified clinician, #19)

Despite these potentials, a major concern of numerical outputs is the limited ability to fully account for patient complexity, diversity, and individuality, particularly in cases of multimorbidity (#8, #9, #12, #13, #14, #16, #17, #18, #19).

I think if you continue to call it “VAD projections” 65%, people are going to poke holes at it. They are gonna try to prove you wrong. This [Decision Support Tool projection] is just what the historical outcomes were. But this guy is different, this guy has his own things that make him special. (Cardiologist, #19)

Clinicians therefore emphasised the need for AI-CDSS integration within electronic health records (EHRs) to access relevant clinical data and contextual information (#1, #2, #5, #9, #13, #15).

However, clinicians identified a fundamental limitation of the system’s applicability to clinical practice: its inability to perceive patients holistically and qualitatively, as they do through ‘clinical gestalt’ (#12, #13, #14, #16, #17) and familiarity with individual patients’ trajectories (#9, #16, #17). As one clinician noted:

I think it’s actually, you also have to know the patient. That means that for me, everything starts from the moment the patient enters through the door, right? And there you can already get quite a lot of information, that is, about character, about stature, about the general condition, what you hear and see and so on. That’s the first impression. Then of course comes the factual (Senior physician, #14)

The need for such qualitative patient assessments was highlighted in one study, where synthetic patient case presentations were met with "long, awkward silence" from interviewees (#19). Nonetheless, clinicians also pointed out that in those situations where they were unfamiliar with patients or unable to communicate, such as during night shifts, AI-CDSSs could be very supportive (#7, #10, #16).

Finally, clinical routines limit the potential of AI-CDSSs: clinicians have insufficient time to fully utilise AI-CDSS features ( #2, #6, #8, #11, #15, #18), data entry needed for AI-CDSS did not align with workflow during patient care (#1, #18), impracticality with medicine dose availability (#9) and (false) alerts were considered too disruptive to clinical tasks (#13, #15, #21).

I just don’t know how many people are going to have time and desire [to read this secondary display], and how useful is that going to be for clinicians when they’re trying to [take] care of the patient (Certified registered nurse anaesthetist, #2)

Clinicians therefore suggested a flexible, adaptive interface with a data display tailored to their needs to prevent information overload in time-constrained environments (#2, #4, #5, #13).

In summary, AI-CDSSs offer several opportunities to improve patient care, including assisting clinicians in prioritising patients, monitoring, accelerating care, communicating and improving task efficiency. However, its potential is limited by challenges, particularly concerning integration into clinical workflows and the complexity and specificity of individual patients.

#### Individual characteristics: competencies and cognitive frameworks

The case study researchers documented individual differences between clinicians in how AI-CDSS recommendations were perceived, attributing them to AI literacy, clinical expertise levels, and experiential anchoring. With AI-CDSSs being recently introduced to healthcare, some clinicians struggled with AI literacy, particularly regarding XAI (#3, #4, #5, #6, #11, #13, #14, #15, #16, #19). As a result, the potential value of AI-CDSS recommendations was often limited by this lack of accurate interpretation (#3, #15, #18, #19).

Clinicians prevalently believed that usage of AI-CDSSs would be most beneficial for junior clinicians (#8, #10, #12, #15, #16, #18, #20) or non-specialists (#15, #20). At times, clinicians expressed such high confidence in their own judgement that they completely disregarded AI-CDSS outcomes (#4, #9, #17, #20). Both #11 and #17 observed a tendency toward confirmation bias, varying in degree across individuals.

Clinicians’ evaluations of AI recommendations are influenced by previous patient encounters, a phenomenon known as “recency bias” (#1, #2), with a particular tendency to be biased toward recent cases (#19, #10). Clinicians showed greater appreciation for AI recommendations in patient cases where they had limited experience (#16, #18). Furthermore, clinicians’ customary practices may conflict with AI recommendations, as clinicians often prefer their own patient management approach (#9, #17, #20).

Lastly, in interviews, clinicians emphasised the prominent role of intuition in patient care, particularly when they were familiar with the patient (#6, #14, #16):

I feel like a lot of times we just kind of know when somebody is, like, not doing well, especially when we have the same patients often like day to day. (Nurse, #16)

Two studies highlighted how senior clinicians’ reliance on intuition in clinical decision-making sets them apart from junior clinicians (#4, #14). This intuition, developed through experience, plays a critical role in making patient management decisions, leading to differences between senior and junior clinicians in how AI-CDSS is used (#14).

In conclusion, the value AI-CDSSs provide can vary among clinicians. Case studies have documented differences in AI literacy, levels of expertise and the resulting need for data-driven assistance, susceptibility to confirmation bias, and lastly, personal experiences with specific patient populations and clinical practices.

#### Perceived Task-Technology-Fit

Clinical interactions with AI-CDSSs varied widely, and this diversity is inherently linked to the nature of the AI-CDSS outcomes; concrete treatment suggestions tend to support a more prominent role for AI-CDSSs in decision-making than general risk scores. Nevertheless, most studies, most notably the research conducted by #17, have documented differences in usage of the same AI-CDSS tool. #14’s analysis of algorithmic decision making highlighted a key distinction between using AI-CDSS as a starting point to guide clinical decisions and using it as a feedback tool to validate decisions.

AI-CDSSs most commonly influenced decision-making indirectly by affecting clinicians’ confidence in a Bayesian-like manner, either reinforcing existing assessments or encouraging reconsideration based on its recommendations (#1, #2, #6, #7, #9, #10, #13, #14, #16, #17, #18, #20, #21). A few illustrative quotes:

Well, it hasn’t changed my current decision, but the basis is much better, and I can easily see that it has helped me. (Electrophysiologist, #10)

So I think that is also a good process that, if you make a deviating recommendation now or come to a deviating result, that you just once again go on the way to look: Did I miss something? And I think that exactly is part of it. (Senior physician, #14)

I am ambivalent about this one. Her [blood pressure] is slightly low. Her heart rate is actually coming down, fluid balance is positive… I think it’s fine. We can do what the AI recommends. (Attending physician Intensive Care Unit, #17)

In a few cases, clinicians completely disregarded AI-CDSS outputs, offering the following reasons: significant deviations from their own clinical assessments (#1, #4, #13, #14, #16), recommendations perceived as illogical (#5, #14, #17), suggestions that were unconventional or outside standard practice (#17, #20), and recognition that the system’s scores failed to account for critical contextual factors (#9, #13, #16, #18). In other instances, while disagreement with AI-CDSS recommendations did not directly influence clinical decisions, it prompted various follow-up action, such as consulting colleagues (#4, #13, #16, #17), ordering tests (#15), performing physical examination (#17), examining patient health records (#16, #20), or increasing patient monitoring (#15). These initiatives, not the AI-CDSS output itself, contributed to their decision-making process, as illustrated by the following quotes:

I think it’s just, as I said before, an additional point that, as I said earlier, in this relatively quick and intuitive process, throws a moment of thought in between, even more when you might be in danger of overlooking something. But I think the decision-making process itself is relatively little influenced by that (Junior physician, #14)

So, the tool helps to reinforce my decision-making. The color-coded recommendations provide a clear visual indication, prompting me to address any discrepancies that may arise between the tool’s suggestions and my own clinical plan. In this case, I delve into additional clinical histories that the tool does not have access to and elucidate the rationale behind my decisions. This process enhances my confidence and guides better decision-making during the clinical visit, which can improve the quality of patient care. (Consultant (senior physician), #20)

In general, two different types of TTF could be observed. First, AI-CDSSs were highly valued for its function as a feedback system, monitoring patient status and alerting to clinical findings to prevent oversights (#2, #6, #9, #13, #14, #16, #20) and signalling potential errors in patient care (#13, #18). Secondly, AI-CDSSs demonstrated value by making more informed decision, for instance by providing alternative viewpoints (#19), consolidating clinical data for clinicians (#1, #10) or uncovering new patterns in data (#2). This expectation led to some clinicians expressing frustration when AI-CDSS features failed to provide any new informative insights (#4, #10, #11, #13, #15, #21).

#### Overview of findings

**Fig. 2:**
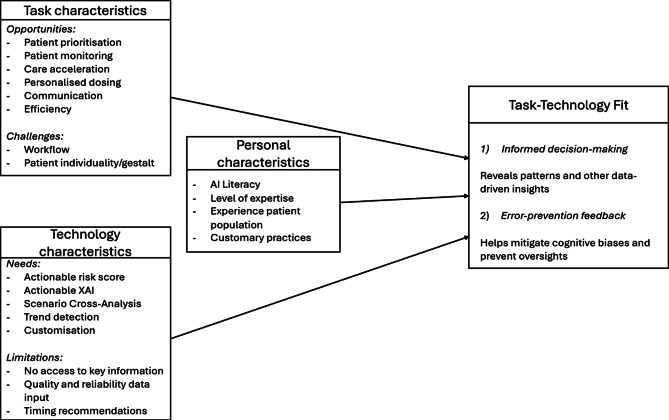
Task-Technology Fit Framework for AI-CDSSs based on analyses of 21 case studies

Figure 2 provides an overview of factors that were repeatedly mentioned in the 21 case studies, organised by task, technology, and individual characteristics. The framework also distinguishes two types of TTF in context of AI-CDSSs: (1) tools for more informed decision-making, and (2) feedback tools for error prevention. While some factors are specific to one type (e.g., scenario cross-analysis), others are shared across both (e.g., customisation).

The figure shows the importance of a contextual understanding in AI-CDSS design. Effective design requires consideration of the intended use and its constraints, and recognising that user factors such as AI literacy and customary practices can influence the fit.

## Discussion

The aim of this review was to describe clinicians’ perspectives when interacting with an AI-CDSS for clinical decision making. We synthesised the qualitative research of published case studies through the lens of the Task-Technology Fit model, highlighting design elements of AI-CDSSs that are misaligned with clinicians’ tasks. In addition, this approach allowed us to explore how AI-CDSSs are used and integrated in clinical decision-making processes.

### Key findings – design implications

Our TTF analysis revealed that the current design of AI-CDSSs is often not fully optimised for clinical tasks. Although none of the included case studies approached their interviews through a TTF lens, we found that clinicians’ evaluations of AI-CDSS features tended to be strongly linked to their relationship to clinical tasks. For example, percentages as outcome were well received in clinical practice when used for patient prioritisation and care coordination communication but tended to cause confusion when applied to individual patient cases where the task relationship was less clear. Similarly, XAI’s data-driven insights were valued primarily to mitigate the risk of a particular event occurring, with several clinicians praising its ability to provide actionable, data-driven insights that enables effective clinical intervention, and less so for understanding the AI system itself. This finding is confirmed by a large multi-method co-design study involving 112 clinicians and developers [57], which identified that clinicians prioritise XAI relevant to the clinical context over purely model-focused explainability. Lastly, where clinicians reported not directly incorporating AI outputs into their decision making, they still valued the system’s ability to raise awareness and encourage reconsideration of their clinical judgement, helping to prevent oversights in patient care.

Clinicians’ suggestions for technical adjustments likewise reflected a TTF perspective. Multiple times, they recommended flexible options for customising interface displays to avoid information overload in time-constrained environments and to address the specific information needs of individual clinicians. In another example, to mitigate some limitations associated with applying AI-CDSSs to their tasks, some clinicians emphasised the importance of integration with EHRs, allowing access to relevant contextual clinical data for more informed decision-making. A recurring theme regarding the design elements was that clinicians want AI-CDSSs to complement their own skills, such as identifying and visualising trends, integrating large amounts of data, and reducing task uncertainty by comparing treatment and diagnosis strategies. These features could assist clinicians in either identifying aspects prone to oversight or by supporting more informed decision making.

### Key findings – theoretical implications

The narrow scope of the TTF model for analysing the use of a technology overlooks critical factors related to utilisation, such as described in Technology Acceptance Model (TAM) and Unified Theory of Acceptance and Use of Technology (UTAUT). UTAUT, in particular, has already been demonstrated to be applicable in the context of clinical decision support systems [58], highlighting the importance of incorporating dimensions such as performance expectancy, effort expectancy, and social influence in the analysis. Omitting these factors may lead to an incomplete understanding of the clinicians’ intention to use AI-CDSSs. Already in the original TTF paper, Goodhue and Thompson [9] extended the TTF model to the Technology-to-Performance Chain to include precursor factors, such as beliefs and social norms, to determine the level of utilisation. Several previous studies have demonstrated that the TTF perspective can be successfully integrated into other models such as TAM [59–62] and UTAUT [63–65], to enable a more holistic analysis of technology adoption.

Specifically, trust is often mentioned in the literature as one of the most important prerequisites for the use of artificial intelligence in healthcare [66]. In most of the studies included in this review, clinicians expressed a desire for peer-reviewed articles from prospective clinical trials validating the efficacy of an AI-CDSS or valued endorsement from colleagues, reflecting the need for social trust. Therefore, this TTF perspective represents a single component within a larger analysis to bridge the gap between development and clinic. Recently, Salimzadeh et al. [67] emphasised the importance of explicitly considering task characteristics when evaluating human-AI decisions. In a non-health care setting, the researchers found that two task-related factors - the level of complexity and of uncertainty - *independently* of their level of trust, significantly influence the extent to which users resort to AI for decision-making, with the level of ‘appropriate reliance’ being negatively affected [67]. Thus, while the concept of trust has been a popular subject in the field of AI to explain utilisation, the importance of other types of analysis such as the TTF should not be overlooked. A study on autonomous buses even found that Task–Technology Fit served as a partial pathway linking trust to behavioural intention to use this type of AI-based technology [65].

Unlike traditional static technologies, AI-CDSSs undergo continuous monitoring and refinement. While this continuous evolution might position TTF as both a temporal assessment and highly situated and therefore less relevant, this adaptive capability actually enhances the importance of understanding task-technology alignment. As we describe in our results, some changes to AI-CDSSs were made based on user feedback aimed at achieving better TTF, such as implementing a three-tier visual risk categorization for patient risk monitoring. One of the included case studies (#21) radically redesigned an already implemented AI-CDSS system after assessing clinical needs [56]. The new model acted as an “early decision support tool” by predicting sepsis progression, visualising uncertainty, and suggesting additional tests to reduce it. Although not yet implemented, participants considered this design far more useful than the previous risk score, as it refined their hypotheses and supported diagnostic decisions by directly addressing their actual information needs during clinical practice. These examples illustrate how, in adaptive AI systems, TTF drives the optimisation process.

### Implications for clinical practice

Frequently, clinicians were sceptical to what degree AI-CDSSs can compete with the clinician’s expertise and intuition, since these systems often fail to account for factors reflecting patient individuality and lack important clinical information that clinicians possess, such as bedside observations. This scepticism could be addressed in two ways. First, education may help clinicians realise that AI-CDSSs can still provide accurate predictions, even without full contextual data. Higher levels of AI literacy are generally associated with a greater willingness to adopt AI and can decrease other sources of scepticism in clinical practice, such as fear of replacement [68]. Second, AI-CDSS design should take into account its limitations in the clinical context by focusing on areas where it can truly add value with information provision. This would require a greater emphasis on enabling better integration of clinicians’ expertise and AI-CDSS outputs, for instance by designing AI-CDSS as an interactive tool. In this role, AI-CDSSs could enhance exploration of different clinical strategies and highlight leverage points for intervention in patient trajectories, thereby broadening the clinician’s perspective (‘umwelt’) and their decision-making ability but not competing with it. Potentially, an interactive design that allows customisation can help safeguard clinicians’ autonomy, as a sense of agency is a core element of autonomy [69]. In the context of XAI, customisation has been already linked to an increased sense of agency [70]. However, Kostick-Quenet [71] warns that option to customise clinical AI can lead to tunnel vision and the overlooking of critical details, and therefore advocates setting limits on the extent of such customisation.

The last notable finding was that clinicians frequently stated that their clinical decisions were not based on the AI-CDSS predictions. While the World Health Organization guidance on *Ethics & Governance of Artificial Intelligence for Health* [72] assumes that AI-CDSSs ‘added little value’ when clinicians disregard AI results, a closer analysis, such as that conducted by some of the studies (e.g. #17) included in this review, reveals a different perspective. In cases where clinicians did not accept the AI results, they often did report taking additional action - such as ordering more tests or consulting colleagues - to re-evaluate their clinical judgment. These actions represent a form of ‘decomposing’ their initial intuitive expertise judgment, a process Dreyfus coined as *deliberative rationality* [73]. According to Dreyfus [73]" the involved intuitive skilled performer deliberates about his behavior in a detached manner that can be called rational because it involves decomposition. But it is his or her intuitive understanding that is examined and decomposed, not the problem itself." (p.56). Thus, AI-CDSSs can improve clinical performance by prompting reflection on the clinician’s own judgment, thereby safeguarding against tunnel vision [73]. This reflective function was already illustrated in the result section by one clinician who noted that an AI-CDSS prompted him to “elucidate the rationale behind my decisions” [55]. This alternative usage of AI-CDSSs requires design and policy considerations to strategically position AI-CDSSs in such a way clinicians can form their initial judgement based on intuition and expertise, before being influenced by an AI-CDSS. It simultaneously calls for a critical reflection on the extent to which the potential of AI-CDSSs to improve clinician performance is being fully realised, when it is solely used for deliberative rationality. Given that a recent multicentre study in colonoscopy revealed early indications of clinician deskilling from ongoing exposure to AI guidance [74], greater consideration in the use and design of AI assistance appears necessary.

### Limitations

The main limitation of our study in assessing the context of any findings was the lack of original interview transcripts and the reliance on the authors’ interpretations from the interview data of the included papers. For example, the extent of clinician AI literacy is largely an interpretation of the authors. While some papers contained more original quotations of clinicians than others, no distinction was made by us between authors’ interpretations and direct quotations, as even the quotations themselves were subject to author selection and potential bias. In addition, differences in interview design and the lack of original transcripts prevented reliable estimation of the frequency of findings. Therefore, we conducted this exploratory analysis with a focus on how technology, task, and individual characteristics interrelate and influence clinicians’ use of AI-CDSSs, without drawing conclusions about specific features, such as whether percentages are an appropriate AI-CDSS output.

Another limitation for the TTF analysis is the diversity of implementation stages of the included AI-CDSSs and thus a varying degree of direct experience with AI-CDSSs. Initially, we included only implemented AI-CDSSs, but the limited number of eligible articles led us to broaden our criteria to include AI-CDSS interactions in simulated settings, which do not fully reflect clinical practice. However, we continued to exclude studies based solely on non-user clinician perspectives, as direct user experience with algorithmic decision-making was considered essential for the TTF analysis. Our choice to include user interaction with non-implemented AI-CDSSs may have resulted in overstatement of some conclusions. We sought to minimise this risk by explicitly indicating the implementation phase for each case study in Table 1. The limited number and heterogeneity of included studies also made it difficult to draw conclusions about how Task-Technology Fit may vary across departments.

Our final limitation relates to the scope of our analysis. We excluded AI-CDSS primarily consisting of computer vision or natural language processing algorithms, as their distinct technological features require a different cognitive task perspective. While this choice allowed us to focus in depth on TTF of tabular systems, it also narrowed the breadth of our analysis. Moreover, as discussed above, TTF does not account for other important determinants of technology use, such as trust or factors from UTAUT and TAM. In addition, although we included studies from different time phases of AI-CDSS implementation, we lacked insight into how the clinician-AI relationship evolves over time. Lastly, although shared decision making is common practice, patients were not included as users of AI-CDDS in these studies.

Therefore, there is a critical need for more qualitative research examining how clinicians’ TTF influences the use of AI-CDSSs. Such work could provide richer insights into how department-specific workflows impact TTF and how AI-CDSS use affects clinical reasoning, as the included case studies exposed already some of the diverse ways AI-CDSSs can be used. Additional TTF analyses should be conducted for image-based and natural language processing AI-CDSS. Future research on algorithmic decision-making should also integrate patients’ perspectives. Finally, as AI-CDSSs have only recently been introduced into clinical practice, their long-term effects, such as potentially decreasing intuition and hindering expertise development, should also be examined. We recommend longitudinal field studies that can specifically document the evolving clinician-AI relationship over time.

## Conclusion

AI-CDSSs have the potential to enhance clinical decision making, yet their success in clinical practice remains limited. Our review of 21 qualitative case studies, analysed through the task-technology fit model, revealed that the design of AI-CDSSs is often not fully optimised for clinical practice. Clinicians commonly struggled with the direct applicability of AI-CDSSs features to their clinical tasks. Furthermore, since AI-CDSSs typically lack access to relevant contextual knowledge held by clinicians, their assessments were often questioned by clinicians for their conclusiveness at the individual patient level. Clinicians primarily valued AI-CDSSs for their ability to generate unique data-driven insights, such as exploring the effects of potential clinical interventions and receiving trend-based information. Our analysis thus highlights design implications that point to the complementary nature of AI-CDSSs in supporting more informed clinical decision making. We also found that AI-CDSSs are being integrated into practice in various ways. Beyond diverse task-specific applications such as patient prioritisation and risk communication, clinicians incorporated the system into their decision-making processes to varying degrees. For some clinicians, it served as a tool for critical reflection, while for others, it was more directly integrated into the decision-making process. Our findings therefore serve as a stepping stone for future case studies seeking to systematically explore the interactions between clinicians and AI. A deeper understanding of these types of integration mechanisms can inform the design of AI-CDSSs that are better aligned with clinical needs, supporting clinicians in their decision-making challenges and contributing to improved patient care.

## Supplementary Information

Below is the link to the electronic supplementary material.

Supplementary Material 1

Supplementary Material 2

## Data Availability

All data analysed were obtained from previously published case studies. The ATLAS.ti project files created for data analysis are available from the corresponding author upon reasonable request.

## Footnotes

1 Defined by [8] as ‘The perceived fit, relevance, or compatibility of the AI for a given clinical setting, user, or consumer, and/or the perceived fit of the AI to address a specific clinical problem’.

2 Defined by [8] as ‘how agreeable, palatable, or satisfactory an AI intervention is perceived’.

## Abbreviations

AI: Artificial Intelligence
AI-CDSS: Artificial Intelligence-based clinical decision support systems
BUC: Blood Utilisation Calculator
EHR: Electronic Health Records
FITT: Fit between Individuals, Task and Technology
ICU: Intensive Care Unit
RCT: Randomised control Trials
RTA: Reflexive thematic analysis
TAM: Technology Acceptance Model
TTF: Task-Technology Fit
UTAUT: Unified Theory of Acceptance and Use of Technology
VAD: Ventricular Assist Device
XAI: Explainable Artificial Intelligence
