# Supplementary material for: Task-Technology Fit of Artificial Intelligence-based clinical decision support systems: a review of qualitative studies
Source: BMC Med Inform Decis Mak. 2025 Oct 28;25:397. doi: 10.1186/s12911-025-03237-8 (PMC12570768; doi:10.1186/s12911-025-03237-8)
Supplement: Supplementary file 1 — Supplementary Material 1 [file 12911_2025_3237_MOESM1_ESM.docx]

# Additional file 1: Search queries

| Dimensions | ("artificial intelligence" OR "ai" OR "machine intelligence" OR "machine learning" OR "deep learning" OR "learning systems" OR "reinforcement learning" OR "supervised learning" OR "unsupervised learning" OR "learning algorithm*" OR "convnet" OR "neural network*") AND (decision* OR CDSS OR CDS) AND ("use case" OR "user case" OR "user experience" OR "user study" OR "user studies" OR "use study" OR "use studies" OR “case study” OR "case studies" OR interview* OR qualitative OR perception* OR "field evaluation" OR "mixed method*" OR "focus group*" ) AND(medic* OR clinic* OR health) NOT (imag* OR radiolog*) |
| --- | --- |
| Web of Science (core collection) | TS=(("artificial intelligence" OR "ai" OR "machine intelligence" OR "machine learning" OR "deep learning" OR "learning systems" OR "reinforcement learning" OR "supervised learning" OR "unsupervised learning" OR "learning algorithm*" OR "convnet" OR "neural network*") AND (decision* OR CDSS OR CDS) AND ("use case" OR "user case" OR "user experience" OR "user study" OR "user studies" OR "use study" OR "use studies" OR “case study” OR "case studies" OR interview* OR qualitative OR perception* OR "field evaluation" OR "mixed method*" OR "focus group*" ) AND(medic* OR clinic* OR health) NOT (imag* OR radiolog*)) |
| Pubmed | ("artificial intelligence"[Title/Abstract] OR ai[Title/Abstract] OR "machine intelligence"[Title/Abstract] OR "machine learning"[Title/Abstract] OR "deep learning"[Title/Abstract] OR "learning systems"[Title/Abstract] OR "reinforcement learning"[Title/Abstract] OR "supervised learning"[Title/Abstract] OR "unsupervised learning"[Title/Abstract] OR "learning algorithm*"[Title/Abstract] OR "convnet"[Title/Abstract] OR "neural network*") AND (decision* OR CDSS OR CDS) AND ("use case"[Title/Abstract] OR "user case"[Title/Abstract] OR "user experience"[Title/Abstract] OR "user study"[Title/Abstract] OR "user studies"[Title/Abstract] OR "use study"[Title/Abstract] OR "use studies"[Title/Abstract] OR "case study"[Title/Abstract] OR "case studies"[Title/Abstract] OR interview*[Title/Abstract] OR qualitative[Title/Abstract] OR perception*[Title/Abstract] OR "field evaluation"[Title/Abstract] OR "mixed method*"[Title/Abstract] OR "focus group*" ) AND(medic*[Title/Abstract] OR clinic*[Title/Abstract] OR health[Title/Abstract]) NOT (imag*[Title/Abstract] OR radiolog*[Title/Abstract]) |
| IEEE Xplore | ("All Metadata":"artificial intelligence" OR "All Metadata":ai OR "All Metadata":"machine intelligence" OR "All Metadata":"machine learning" OR "All Metadata":"deep learning" OR "All Metadata":"learning systems" OR "All Metadata":"reinforcement learning" OR "All Metadata":"supervised learning" OR "All Metadata":"unsupervised learning" OR "All Metadata":"learning algorithm" OR "All Metadata":"learning algorithms" OR "All Metadata":"convnet" OR "All Metadata":"neural network" or OR "All Metadata":"neural networks") AND ("All Metadata":decision* OR "All Metadata":CDSS OR "All Metadata":CDS) AND ("All Metadata":"use case" OR "All Metadata":"user case" OR "All Metadata":"user experience" OR "All Metadata":"user study" OR "All Metadata":"user studies" OR "All Metadata":"use study" OR "All Metadata":"use studies" OR "All Metadata":“case study” OR "All Metadata":"case studies" OR "All Metadata":interview* OR "All Metadata":qualitative OR "All Metadata":perception* OR "All Metadata":"field evaluation" OR "All Metadata":"mixed method" OR "All Metadata":"mixed methods" OR "All Metadata":"focus group" OR "All Metadata": "focus groups") AND ("All Metadata":medic* OR "All Metadata":clinic* OR "All Metadata":health) NOT ("All Metadata":imag* OR "All Metadata":radiolog*) |

*Table 2: Search queries used for literature search per database*

# Additional file 2: coding list

| **Code** | **Description** | **Example(s)** |
| --- | --- | --- |
| Individual_AI literacy | Degree to which understand how artificial intelligence functions | “Only three participants understood the approach of example-based explanations which was based on influential patients.” (#3)  “P9 had a false interpretation of a confidence explanation and stated that “44% certainty in a diagnosis is a good value. “ (#11) |
| Individual_clinical skills | Degree to which clinicians have clinical experience | “This system can help junior doctors learn from the experience of senior doctors in a more interactive way instead of plain text [in] the textbook.” (#8) |
| Individual_confidence | Degree to which clinicians are confident in their clinical judgement | “I would say that I'm as good or even better than the system. I don't feel the need to rely on it; I'll just do what I do. We are all trained endocrinologists, so we trust our judgment because that has been our bread and butter for many years..” (#20 ) |
| Individual_intuition | Clinicians relying on intuition for clinical judgement | “Nurses have instinct and there are visual cues that an [artificial intelligence] could never capture...” (#6) |
| Individual_patient population | Clinicians experience with a particular patient population | “Other participants felt that the scores reflected their experience with patients with COVID-19: “Those numbers were relatively reasonable to what I have seen” “ (#1)  “I’m looking at 100 [patients] and overweighting the last three I saw.” (#19) |
| Individual_clinical practices | Individual approaches to delivering care | “Two participants found the AI’s recommendations “a bit fuid aggressive” (P2) at times, particularly because they perceived that many clinicians overuse fuids: “I’ve seen it in ICU where we’re just like bolusing them blindly. And the next thing you know, they’re puffy like the Michelin man” (P1). “ (#17 ) |
|  |  |  |
| Technology _ modifiability | Degree to which AI-CDSSS output is regarded modifiable ( capable of being effectively acted upon) | “… mentioning the AI could change its decision if [the] age was 29 does not consider as a useful explanation in our setting. … I mean, we all know that. … Explanations should be smart enough.” (#11) |
| Technology _scenarios | Predictions generated for different situations to illustrate the potential effects of various actions or interventions | “I can see me using this but with more information. I need to know if you put a kind of chemotherapy, something will happen. If you use this type of therapy, you will have better outcomes. [We] need to know something more.”–Participant 2 (#3) |
| Technology_customisation | Users being able to alter AI-CDSSs features | “I think it's important that you can yourself let your prioritisation flow into it, that it's possible for you, that it can also be changed. That you don't somehow get a score where you had no possibility to influence it. That one simply makes use of the great computing power and also the better concentration ability of many variables, but says oneself: the variable is most important to me, then this one comes, then this one comes. And that you can possibly also say: okay, the result surprises me. Now I turn these two variables around again, because they are almost equally important” (#14) |
| Technology_data input | The data that AI-CDSSs relies on for its output | “One participant liked that the application did not “need biochemical parameters,” which rendered it more “useful in [the] ED setting” [Participant #22, foundation physician], as it negated the need to wait for the results of blood tests and allowed for more rapid quantification of the patient’s risk. (#1) |
| Technology_data integration | Type of data integrated into AI-CDSS interface | “I like having the guidelines built-in so that you know when you’re doing something that is, um, the, that is the guideline or evidence based. And, you know, when you are deviating from that and therefore hopefully have a good reason for it and are at least cognizant of the fact that you're deviating.” (#4) |
| Technology_trends | AI-CDSS outputs that highlight temporal changes in patient data | “And you know what else would be helpful, instead of a dash, to actually have an arrow, like a sideways arrow. Just to show that, ‘Okay, this one’s stable’ or ‘This one’s not increasing or decreasing.’” - Participant 2 (#5) |
| Technology_output | Type and nature of AI-CDSS recommendation | “I think if you continue to call it “VAD projections” 65%, people are going to poke holes at it. They are gonna try to prove you wrong. This [DST projection] is just what the historical outcomes were. But this guy is diferent, this guy has his own things that make him special. “ (#19) |
| Technology_timing | Stage in a patient's trajectory when AI-CDSS outputs are delivered | “It gets tough to treat something ahead of time, because if someone's doing okay right there right now, if you want to give them a treatment that could potentially cause them harm, that might … give us cause to pause to sort of be pre-emptive about that.” (#6) |
| Technology_XAI | Explainable AI | “it's very nice to see that the algorithm reacts on the same parameters that I've discovered myself ... So it's nice to see that I agree with it. You could say that it’s supporting and it's safe to know, that it also says there was something here. “ (#10) |
|  |  |  |
| Task_communication | Being able to facilitate communication between colleagues and/or patients | “The complications risk prediction feature stands out as particularly beneficial to me. For example, it provides an alert regarding the risk of hypoglycemia. When the risk level is classified as moderate or high, this information helped me better persuade patients to consider specific treatments or to improve their compliance with the recommended approach. “ (#20) |
| Task_complexity | The degree of task complexity | “I think I would always accept AI if the amount of data is just not analysable and capturable for me” (#14) |
| Task_efficiency | (Potential) effects on reducing cognitive effort and simplifying task execution | “Earlier, we used to decide how many units like haphazardly, but with BUC, I like that it does part of my thinking. Well, I would say it’s easier because now I don’t have to think as it tells me how many units of blood, I need to give a patient.” (#4) |
| Task_care acceleration | Enabling faster medical decision-making, facilitating timely interventions and care escalation | “The bar representing the range of delirium risk helps us to identify patients at the border to another risk group.” (#7)  “Due to the delirium prediction application, we were already able to prevent the sliding into a strong delirium with simple interventions.” (#7) |
| Task_objectifying | The need to objectify medical decision making | “Perhaps, the advantage of the algorithm is that it is not influenced by what the individual clinician has experienced within the last month, and in this way helping to make more uniform conclusions. [Interview, Electrophysiologist #5]” (#10)  “When I really like this patient, really want to help him or her, it sometimes helps to get a more factual view.” (#19) |
| Task_patient priorisation | Being able to rank patients based on urgency or risk, guiding clinicians to allocate attention and resources more effectively between patients | “The EWS triggers my mind to investigate a chart and see what's going on and identifies patients to focus on, evaluate first, and decide if I need to intervene. (RRT)” (#13) |
| Task_patient specificity | The need to take into account unique characteristics and needs of an individual patient | “I feel like a lot of times we just kind of know when somebody is, like, not doing well, especially when we have the same patients often like day to day. [RN 1] You need those people to look at those numbers that are like patient’s tachycardic, heart transplant to say, “yeah, that’s abnormal, but it is normal.” And in a sense, you can’t really computerize that stuff. So that’s why a clinician’s judgment is so important...you need someone to be thinking, like, what do these numbers actually mean?” (#16) |
| Task_uncertainty | The degree of task uncertainty | “I’m like doing night coverage, so I don’t know the patients as well, so maybe I would, in that setting, be more reliant on a tool like that. [Physician 4]” (#16) |
| Task_variability | The degree of heterogeneity in the medical conditions on which AI-CDSSs are applied | “The guidelines are changing, and so the data itself may change” [Participant #15, consultant] (#1)  “ I’d want to know a bit more about how it was developed, and so let’s say the data that CONCERN was trained on was exclusively ICU sepsis and organ failure, mortality, all-cause mortality, let’s say...then I would say this tool is only generalizable to the ICU setting, for example.” [Physician 3] (#16) |
| Task_workflow | Workflow characteristics in which the AI-CDSS operates, determining its potential | “ I’m not saying that systems like this aren’t smart, but I just feel like so much of it depends on what’s going on in that moment. And a lot of times, you know, our documentation isn’t always like right up to date with what’s going on at the moment. “ (#16) |
|  |  |  |
| Utilisation_discordance | Reasoning and actions when the AI-CDSS output and clinical judgement are not aligned | “ If I looked at the tool and it said to me ‘okay, she’s got a 4% chance of mortality’, but I look at the patient at the end of the bed and they appear incredibly frail, in that instance my judgement would overrule the application’s prediction. “[Participant #18, registrar] (#1)  “If the AI was disagreeing with me, what I would do is walk into the room, do a leg raise, do a ultrasound... and then based  on that information, I would decide how much volume to give.” (#17) |
| Utilisation_feedback | The use of AI-CDSSs to provide feedback after a clinical judgement | “So I think that is also a good process that, if you make a deviating recommendation now or come to a deviating result, that you just once again go on the way to look: Did I miss something? And I think that exactly is part of it” (#14) |
| Utilisation_integration decision making | The extent to which AI-CDSS recommendation can be integrated in clinical reasoning processes | “Because the system provides several diagnosis suggestions, it is a huge help for expanding our thoughts.  In particular, the AI-CDSS system could facilitate the diagnosis of uncommon disease for new patients. I do feel it greatly improves our work.” (#18) |
| Utilisation_value | In what ways AI-CDSSs augment decision making or negatively impacts decision making | “I'd call it a second set of eyes and ears for the clinician…Clinical judgment supersedes any tool, but it's there to help you pick up potential septic patients, and that's one component, but the other component is it can help keep you on track with the core measures” (#6)  “I think there are a lot of people frankly that will quickly default to having a tool tell them what to do and stop assessing, and I hope that's not true, but I've seen it happen” (#6) |

*Table 3: The coding list is organised around four main categories from the Task-Technology Fit framework - Task, Technology, Individual and Utilisation - with sub-themes identified through inductive coding.*
